# Factor H autoantibody is associated with atypical hemolytic uremic syndrome in children in the United Kingdom and Ireland

**DOI:** 10.1016/j.kint.2017.04.028

**Published:** 2017-11

**Authors:** Vicky Brocklebank, Sally Johnson, Thomas P. Sheerin, Stephen D. Marks, Rodney D. Gilbert, Kay Tyerman, Meredith Kinoshita, Atif Awan, Amrit Kaur, Nicholas Webb, Shivaram Hegde, Eric Finlay, Maggie Fitzpatrick, Patrick R. Walsh, Edwin K.S. Wong, Caroline Booth, Larissa Kerecuk, Alan D. Salama, Mike Almond, Carol Inward, Timothy H. Goodship, Neil S. Sheerin, Kevin J. Marchbank, David Kavanagh

**Affiliations:** 1 National Renal Complement Therapeutics Centre, Newcastle University, Newcastle upon Tyne, UK; 2 Great North Children's Hospital, Sir James Spence Institute, Royal Victoria Infirmary, Newcastle, UK; 3 Great Ormond Street Hospital for Children NHS Foundation Trust, London, UK; 4 University Hospital Southampton NHS Foundation Trust, Southampton, UK; 5 Leeds Teaching Hospitals NHS Trust, Leeds, UK; 6 The Department for Paediatric Nephrology & Transplantation, The Children's University Hospital, Dublin, Ireland; 7 Department of Paediatric Nephrology, Royal Manchester Children’s Hospital, Central Manchester University Hospitals NHS Foundation Trust, Manchester Academic Health Science Centre, Manchester, UK; 8 University Hospital of Wales, Cardiff, Wales; 9 Guy’s and St Thomas’ NHS Foundation Trust, London, UK; 10 Birmingham Children’s Hospital NHS Foundation Trust, Birmingham, UK; 11 UCL Centre for Nephrology, Royal Free London NHS Foundation Trust, Rowland Hill Street, London, UK; 12 Southend University Hospital, Prittlewell Chase, Westcliff-on-Sea, UK; 13 Department of Paediatric Nephrology, Bristol Royal Hospital for Children, Bristol, UK; 14 Institute of Cellular Medicine, Newcastle University, Newcastle upon Tyne, UK

**Keywords:** acute kidney injury, atypical hemolytic uremic syndrome, complement, factor H autoantibodies, thrombotic microangiopathy

## Abstract

Factor H autoantibodies can impair complement regulation, resulting in atypical hemolytic uremic syndrome, predominantly in childhood. There are no trials investigating treatment, and clinical practice is only informed by retrospective cohort analysis. Here we examined 175 children presenting with atypical hemolytic uremic syndrome in the United Kingdom and Ireland for factor H autoantibodies that included 17 children with titers above the international standard. Of the 17, seven had a concomitant rare genetic variant in a gene encoding a complement pathway component or regulator. Two children received supportive treatment; both developed established renal failure. Plasma exchange was associated with a poor rate of renal recovery in seven of 11 treated. Six patients treated with eculizumab recovered renal function. Contrary to global practice, immunosuppressive therapy to prevent relapse in plasma exchange–treated patients was not adopted due to concerns over treatment-associated complications. Without immunosuppression, the relapse rate was high (five of seven). However, reintroduction of treatment resulted in recovery of renal function. All patients treated with eculizumab achieved sustained remission. Five patients received renal transplants without specific factor H autoantibody–targeted treatment with recurrence in one who also had a functionally significant *CFI* mutation. Thus, our current practice is to initiate eculizumab therapy for treatment of factor H autoantibody–mediated atypical hemolytic uremic syndrome rather than plasma exchange with or without immunosuppression. Based on this retrospective analysis we see no suggestion of inferior treatment, albeit the strength of our conclusions is limited by the small sample size.

Atypical hemolytic uremic syndrome (aHUS) is commonly a consequence of complement dysregulation^1^ and is characterized by the triad of microangiopathic hemolytic anemia, thrombocytopenia, and acute kidney injury.^1^ It is rare, with an incidence of 0.42 per million in the United Kingdom,^2^ but associated with significant morbidity and mortality.

aHUS is associated with mutations in genes encoding complement regulatory proteins, complement factor H (*CFH*), complement factor I (*CFI*), and membrane cofactor protein (*CD46*)^1^ and in genes encoding the complement components complement factor B (*CFB*) and C3 (*C3*).^1^ For all of these complement mutations, penetrance is incomplete and influenced by genetic modifiers in addition to environmental triggering events.^1^

aHUS is also associated with acquired complement dysregulation, occurring consequent to the development of autoantibodies directed against complement factor H (FH)^3^ and factor I.^4^ Anti-FH–associated aHUS predominantly presents in childhood.^3^ The proportion of children with aHUS who have anti-FH autoantibodies has been reported as 5% to 25% in European cohorts^5^ and as high as 56% in a large Indian cohort.^6^ Anti-FH autoantibodies have been shown to be directed against different epitopes, in some reports exclusively^7^, ^8^ but in others predominantly^9^, ^10^, ^11^ located at the C-terminal of FH; a polyclonal response to both N and C termini has also been reported,^10^, ^12^ and functional analyses have demonstrated disruption of complement regulation by multiple mechanisms.^12^

A strong association has been observed between anti-FH autoantibodies and a homozygous deletion of *CFHR1* and *CFHR3,* which encode complement FH–related proteins 1 and 3. A deletion encompassing *CFHR1* and *CFHR4* has also been reported in anti–FH- associated aHUS, suggesting that it is the absence of FH-related protein 1 that is most important in the development of FH autoantibodies,^1^, ^9^, ^12^, ^13^, ^14^, ^15^, ^16^, ^17^, ^18^, ^19^ and homozygous deletion of *CFHR1* has been identified in 79% to 89% of affected individuals.^5^

Recent international consensus recommendations on the management of aHUS highlight the uncertainty regarding optimal management in patients with anti-FH autoantibodies.^20^ In the United Kingdom and Ireland, our management regimen, in contrast to practice in other countries, has not incorporated immunosuppression. We report here our experience of the management of pediatric FH autoantibody–associated aHUS.

## Results

### Patient details

**Figure 1:**
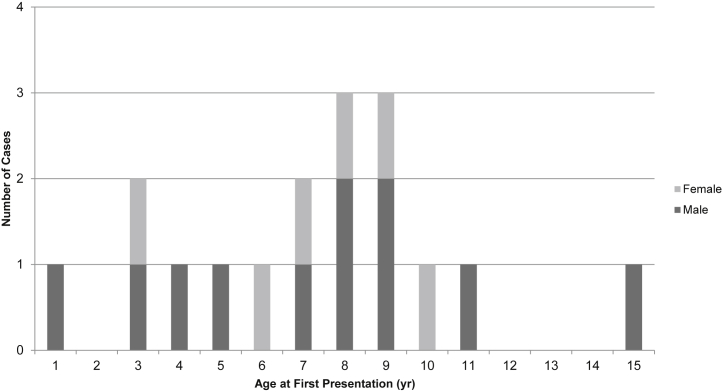
**Age and sex at first presentation of factor H autoantibody-associated atypical hemolytic uremic syndrome.** The median age at presentation was 8 years (range, 1–15 years). There was a male predominance: 65% male, 35% female.

A total of 175 children younger than 16 years of age from the United Kingdom and Ireland who were referred to the UK National aHUS center between 2000 and 2015 were examined for FH autoantibodies. Twenty-two children (13%) had positive FH autoantibody results; 17 of 22 children (9.7% of the total cohort) had titers higher than the international standard^21^ (100 relative units) on serum samples obtained at the time of the initial presentation and were included in this study (mean follow-up was 6 years, 5 months; range, 7 months to 13 years, 7 months). Five of 22 children did not meet the inclusion criteria (details in Supplementary Methods). In all patients, a clinical diagnosis of aHUS was made based on the presence of microangiopathic hemolytic anemia, thrombocytopenia, and acute kidney injury and the absence of Shiga toxin and secondary causes.^2^ In 7 patients, a renal biopsy was performed that demonstrated thrombotic microangiopathy. The median age at presentation was 8 years (range, 1–15 years), and there was a male preponderance (male:female ratio = 11:6) (Figure 1).

### Clinical presentation

Clinical features at the time of presentation are shown in Table 1. Prodromal gastrointestinal symptoms were commonly observed, including abdominal pain (9/16 patients), vomiting (9/16 patients), and diarrhea (8/16 patients). Infection was reported as a triggering event in 7 of 17 patients. Extrarenal manifestations were reported in 8 of 16 patients and included central nervous system involvement, hepatitis, and pancreatitis. The serum creatinine and platelet values at presentation are shown in Supplementary Figure S1.

**Table 1:** Clinical features at presentation

Clinical feature	Number of patients	
Age, yr (*N* = 17)	Median: 8 yr; range: 1–15 yr	
Sex (*N* = 17)	Male: *n* = 11	Female: *n* = 6
Prodrome (*N* = 16)	Abdominal pain	*n* = 9^b^
	Vomiting	*n* = 9^b^
	Diarrhea	*n* = 8^b^
	Fever	*n* = 4
	Other	a
Clinical features (*N* = 16)	Hematuria	*n* = 12
	Proteinuria	*n* = 13
	Oligoanuria	*n* = 10
	Hypertension	*n* = 9
	Edema	*n* = 3
Triggering event (*N* = 17)	Infection	*n* = 7
	None identified	*n* = 10
Extrarenal manifestations (*N* = 16)	None	*n* = 8
	Seizures	*n* = 3
	Hepatitis	*n* = 2
	Epistaxis	*n* = 2
	Pancreatitis	*n* = 1
	Altered consciousness	*n* = 1

Laboratory features at presentation	Mean (range)
Hemoglobin, g/dl (*N* = 17)	6.6 (4–9.1)
Platelet count, ×10^9^ (*N* = 17)	52 (9–134)
Creatinine, μmol/l (*N* = 17)	331 (61–1131)
Lactate dehydrogenase, U/L (*N* = 16)	4460 (887–17,584)
Antinuclear antibodies (*N* = 12)	1/12 positive 1:40

a Other prodromal symptoms reported in ≤2 patients: headache, malaise, cough, visible hematuria, petechiae, and flulike illness.

b Twelve patients had at least 1 gastrointestinal symptom (abdominal pain, vomiting, diarrhea).

### Complement analysis (*N* = 17)

**Figure 2:**
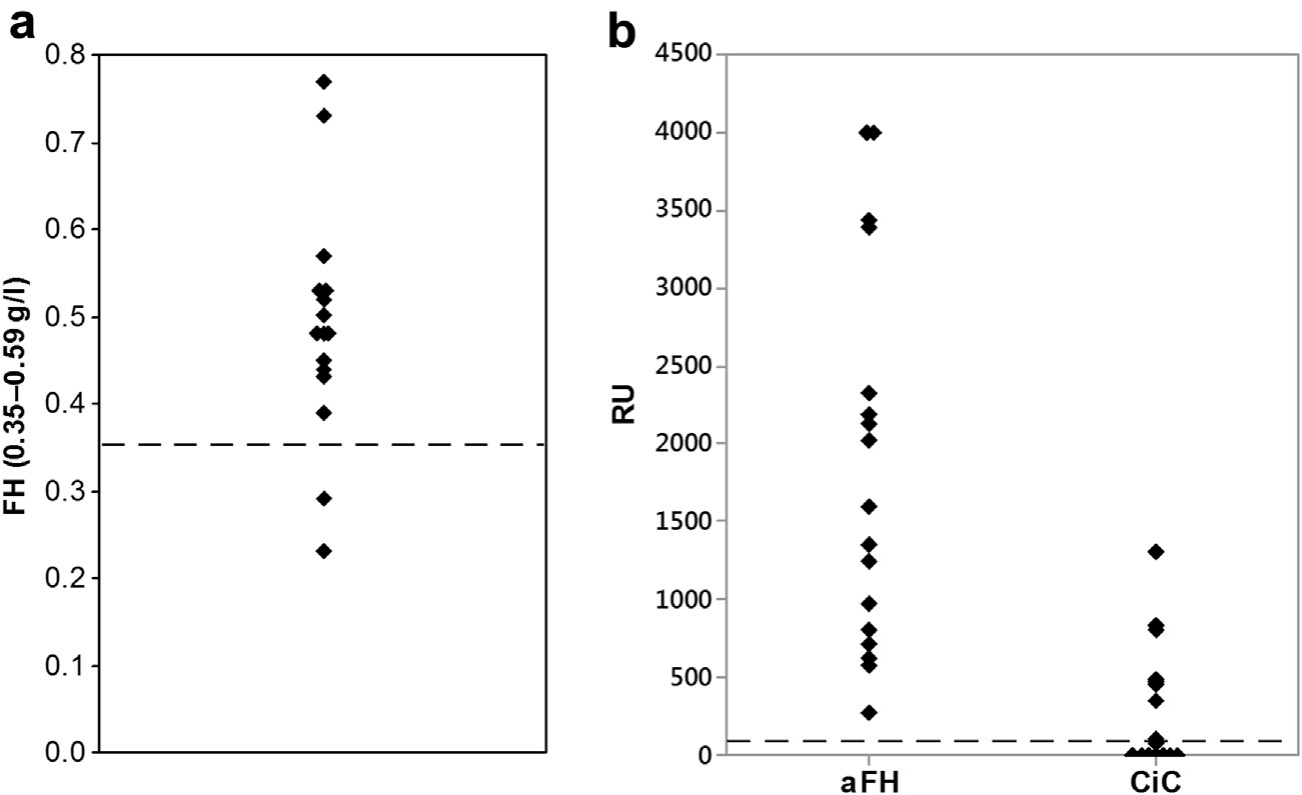
**(a) Initial titers of factor H autoantibody and circulating immune complexes of factor H/autoantibody.** In all patients the initial FH autoantibody titer was above the international consensus positive threshold of 100 relative units (dashed line). In some, but not all patients, circulating immune complexes were detected. (**b**) Initial factor H level. The dashed line represents the lower limit of the normal range. Fifteen patients (88%) had a normal FH level. Two patients (12%) had a low FH level, and neither had a *CFH* rare genetic variant. The patient with a rare genetic variant in *CFH* had a normal FH level. aFH, FH autoantibody; CiC, circulating immune complexes; FH, factor H; RU, relative units.

Initial complement levels are shown in Table 2 and Supplementary Figure S2 (*N* = 17). Thirteen patients had normal C3 levels and 12 had normal C4 levels; 3 patients (patients 2, 20, and 24) had both low C3 and low C4. Two patients (patients 5 and 15) had low FH levels (Figure 2a), and neither was found to have a rare genetic variant in *CFH*. No patients had abnormal levels of CD46 or factor I.

**Table 2:** Initial complement antigenic levels: C3, C4, CD46, and factor H (FH), factor I (CFI)

Patient^a^	C3(0.68–1.38 g/l)	C4 (0.18–0.6 g/l)	CD46	FH (0.35–0.59 g/l)	FI (38–58 mg/l)
2	0.57	0.15	Not tested	0.44	70
4^b^	0.89	0.3	Normal	0.52	41
5^b^	0.92	0.2	Not tested	0.29	Not tested
6^b^	1.08	0.36	Not tested	0.5	74
10	1.06	0.13	Not tested	0.45	68
12^b^	1.02	0.19	Normal	0.57	62
14	0.65	0.31	Normal	0.43	41
15	0.77	0.29	Normal	0.23	84
16	0.95	0.51	Normal	0.53	42
17	1.06	0.24	Normal	0.53	44
18	1.1	0.18	Normal	0.48	52
19^b^	1.23	0.52	Normal	0.48	70
20	0.51	0.16	Not tested	0.48	59
21	1.61	0.29	Normal	0.77	73
22^b^	1.22	0.2	Normal	0.73	78
23	0.91	0.16	Not tested	0.39	65
24	0.61	0.14	Normal	0.51	62

FH, factor H; FI, factor I.

a Identification numbers for patients 2, 4, 5, 6, 10, and 12 correspond to those in a previous publication.^9^

b Indicates patients in whom established renal failure developed.

### Anti-FH autoantibodies (*N* = 17)

**Figure 3:**
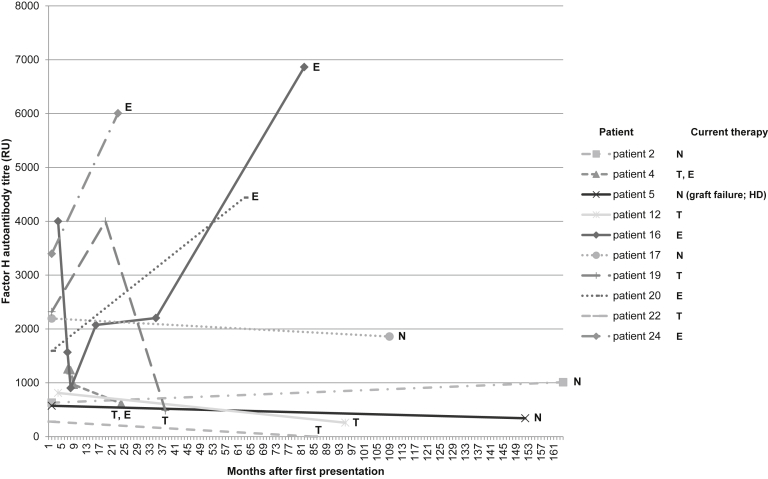
**Changes in factor H autoantibody titers over time for 10 patients.** In 10 patients, anti–FH autoantibody titer measurements at multiple time points up to 163 months after the first presentation were available. In 1 patient, the titer fell below the international standard. The maintenance treatment received by each patient is shown. E, on eculizumab; FH, factor H; HD, hemodialysis; N, no specific maintenance treatment; T, transplanted (immunosuppressed).

The anti-FH autoantibody titers at presentation are shown in Figure 2b (range, 277–4000 relative units; median, 1594 relative units). In 9 of 17 patients, circulating FH/autoantibody immune complexes were detected, and these patients had lower C4 levels (Supplementary Figure S3). In 10 patients, anti-FH autoantibody titer measurements at multiple time points up to 163 months after the first presentation were available (Figure 3). In only 1 patient did the FH autoantibody titer decrease to less than the international standard. In 3 patients treated with eculizumab only, the titer increased. In 5 patients who received immunosuppression in association with current (*n* = 4) or previous (*n* = 1) transplantation, 1 of whom was also treated with eculizumab, the titers decreased, but 4 of 5 remained higher than the international standard. In 2 patients (patients 2 and 17) who did not receive eculizumab or immunosuppression and recovered renal function and remain in remission, the titers remain positive 12 (patient 2) and 9 (patient 17) years after the first presentation.

**Figure 4:**
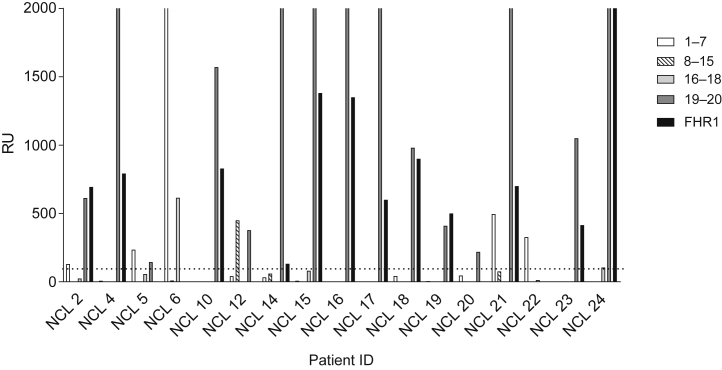
**Autoantibody reactivity with short factor H fragments.** Autoantibody binding to FH fragments (corresponding to SCRs 1–7, 8–15, 16–18, and 19–20) and a FH–related protein 1 fragment (SCR 4–5). In 31% of patients the antibodies were polyclonal. In 69% of patients the antibodies were monoclonal, and in 91% of these the binding was to SCRs 19–20. Two of the 3 patients with 2 copies of *CFHR1* had antibodies that bound predominantly to SCRs 1–7. FHR1, factor H–related protein 1; ID, identification; NCL, Newcastle; RU, relative units; SCR, short consensus repeat.

The binding epitopes of the anti-FH autoantibodies were determined (Figure 4 and Supplementary Table S1). In 15 of 17 patients, the antibodies bound to the C-terminal; 14 of these 15 patients had homozygous deletion of *CFHR1,* whereas the 2 patients (patients 6 and 22) with antibodies that did not bind to the C-terminal did not have a deletion of *CFHR1*. In 12 of 15 patients with antibodies that bound to FH short consensus repeats 19 and 20, there was also positive binding to the C-terminal short consensus repeats 4 and 5 of FH-related protein 1. In 2 patients, the autoantibodies apparently cross-reacted with FH-related proteins 2 through 5 (Supplementary Table S2), but this was not confirmed on Western blotting (not shown), suggesting this interaction was nonspecific or low-affinity binding.

### Genetic analysis (*N* = 17)

The results of the genetic analysis are shown in Table 3. In 7 of 17 patients, a coexisting rare genetic variant in a known aHUS-associated gene was identified: *CFI* (*n* = 4), *CFH* (*n* = 1), *CD46* (*n* = 1), and *C3* (*n* = 1); some have previously been demonstrated to be functionally significant.^1^ In 3 of these 7 patients (patients 4, 5, and 12), established renal failure (ERF) developed.

**Table 3:** Genetic analysis

Patient^a^	Rare genetic variant	Copies of *CFHR1*	Copies of *CFHR3*	Copies of *CFHR4*
2	NMD	0	0	2
4^b^	*CFI* c.1216C>T p.(Arg406Cys)	0	1	1
5^b^	*C3* c.1898A>G p.(Lys633Arg)	0	0	2
6^b^	NMD	2	2	2
10	*CFI* c.1534+5G>T	0	1	1
12^b^	*CFH* c.2850G>T p.(Gln950His)	0	0	2
14	*CFI* c.1456T>C p.(Trp486Arg)^c^	0	0	2
15	NMD	0	0	2
16	*CFI* c.859G>A p.(Gly287Arg)	0	0	2
17	*CD46* c.919A>C p.(Thr307Pro)	0	0	2
18	NMD	0	0	2
19^c^	NMD	2	2	2
20	NMD	0	1	1
21	NMD	0	0	2
22^c^	NMD	2	2	2
23	NMD	0	0	2
24	NMD	0	0	2

NMD, no mutation detected.

Rare genetic variants (defined as observed frequency of <1%, and resulting in a nonsynonymous amino acid substitution or potentially affecting a splice site) identified following mutation screening of the *CFH*, *CD46*, *CFI*, *CFB*, *C3* and *DGKE* genes, and number of copies of *CFHR1, CFHR3* and *CFHR4* as determined by MPLA analysis.

a Identification numbers for patients 2, 4, 5, 6, 10, and 12 correspond to those in a previous publication.^9^

b Homozygous variant (all other variants are heterozygous).

c Indicates patients in whom established renal failure developed.

Fourteen of 17 patients had homozygous deletion of *CFHR1*; in 11 patients, there was homozygous deletion of *CFHR1*/*CFHR3,* and in 3 patients, there was a combined deletion of *CFHR1*/*CFHR3* and *CFHR1*/*CFHR4*. In the total cohort of children from the United Kingdom and Ireland who were negative for anti-FH autoantibodies and underwent full genetic analysis, homozygous deletion of *CFHR1* was identified in 11% (14/122); *CFHR1* deletion was significantly associated with FH autoantibodies in aHUS (*P* < 0.01). Three patients had 2 copies of *CFHR1*, *CFHR3*, and *CFHR4* (patients 6, 19, and 22); ERF developed in all of them.

### Treatment and evolution of the first episode (*N* = 17)

**Figure 5:**
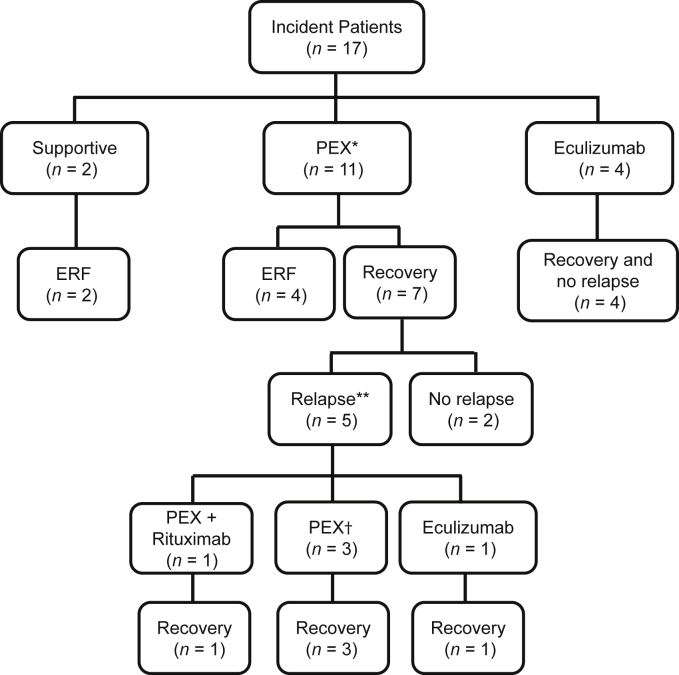
**Evolution of first episode according to treatment.** In children managed supportively, ERF did not develop; treatment with PEX was associated with a high rate of relapse if ERF did not occur, and ERF did not develop in any patient treated with eculizumab or no patient experienced relapse. No patient was treated with immunosuppression at the initial presentation. *Includes PEX alone (*n* = 8), PEX plus i.v. IgG (*n* = 2), PEX plus corticosteroids (*n* = 1). **Defined as recurrence >1 month after presentation and >15 days after disease remission. ^†^One patient (patient 10) experienced multiple relapses and was maintained on regular PEX. ERF, established renal failure; PEX, plasma exchange.

Eight of 17 patients required dialysis within the first week of presentation; ERF developed in 2 of 8 patients, but 5 of 8 patients were dialysis dependent for less than 1 week and fully recovered renal function, and 1 of 8 patients (patient 24, treated with eculizumab) was dialysis dependent for 5 weeks before recovery of renal function (Supplementary Figure S4). Figure 5 summarizes the treatment and evolution of the first episode. Two of 17 patients were managed supportively, and ERF developed in both. Eleven of 17 patients were treated with plasma exchange (PEX), either alone (8 of 11 patients) or in combination with i.v. IgG (2 of 11 patients) or corticosteroids (1 of 11 patients). Of the patients treated with PEX (*n* = 11), ERF developed in 4; 7 recovered renal function, 5 of whom subsequently experienced relapse. Four of 17 patients were treated with eculizumab at the first presentation and continue on this therapy, and none have experienced any relapses. The estimated glomerular filtration rate (eGFR) at the most recent follow-up for all patients is shown in Supplementary Table S3. No patients were treated with immunosuppression at the first presentation. ERF developed in 6 of 17 patients and did so in all following their initial presentation (Supplementary Figure S5). No patients died.

### Relapse (*n* = 5)

Five of 17 patients experienced relapse (Table 4). All had been treated with PEX at the first presentation. Four of 5 patients experienced a single relapse 43 to 1254 days after the initial presentation; 2 were treated with PEX alone, 1 with PEX plus rituximab (patient 21, 16 years 6 months of age at the time of relapse and was managed by the adult nephrology service), and 1 was treated with eculizumab. Three patients have an eGFR of >60 ml/min per 1.73 m^2^, and 1 patient has an eGFR of 53 ml/min per 1.73 m^2^, and none has experienced further relapse. One of 5 patients experienced multiple relapses and was treated with regular PEX for 7 years. PEX was withdrawn in 2012 with no further relapses; 1 year after PEX withdrawal (at last follow-up under pediatric care), the eGFR was >60 ml/min per 1.73 m^2^.

**Table 4:** Incidence and treatment of relapse (*N* = 5)

Patient	Duration of PEX at first presentation	Time to relapse^a^ after first presentation	Treatment of relapse	Outcome
2	PEX × 32 exchanges	88 days	PEX	Creatinine 100 μmol/l eGFR >60 ml/min per 1.73 m^2^ No further relapse
10	PEX × 30 exchanges	Multiple relapses	PEX^b^	Creatinine 83 μmol/l eGFR >60 ml/min per 1.73 m^2^ Relapse-free since 2012
16	PEX × 8 mo	1254 days	Eculizumab	Creatinine 86 μmol/l eGFR 53 ml/min per 1.73 m^2^ No further relapse
18	PEX × 5 exchanges	43 days	PEX	Creatinine 89 μmol/l eGFR >60 l/min per 1.73 m^2^ No further relapse
21	PEX × 15 exchanges	266 days	PEX + rituximab	Creatinine 87 μmol/l eGFR >60 ml/min per 1.73 m^2^ No further relapse

eGFR, estimated glomerular filtration rate; PEX, plasma exchange.

The 2 patients treated with PEX at first presentation who recovered renal function and did not experience relapse were patient 17 (PEX twice weekly for 2 months) and patient 23 (PEX × 6 exchanges); both patients have an eGFR of >60 ml/min per 1.73 m^2^.

eGFR by Schwartz formula for patients younger than 18 years of age at last follow-up (patients 10, 16, and 18) and by an abbreviated Modification of Diet in Renal Disease equation for patients older than 18 years of age at the last follow up (patients 2 and 21).

a Relapse defined as recurrence >1 month after presentation and >15 days after disease remission.

b Patient 10 experienced multiple relapses and was PEX dependent between 2004 and 2011 (PEX every 4 weeks), but has not experienced further relapse since PEX was withdrawn in 2012.

### Outcome of renal transplantation (*n* = 6)

Five of the 6 patients in whom ERF developed received renal transplants (1 patient received 2 transplants) (Table 5). One patient has been ineligible for transplantation due to medical problems. In 1 patient (patient 4), who has a functionally significant *CFI* mutation in addition to FH autoantibodies, a donor after cardiac death transplant resulted in primary nonfunction. Six months later, a living related ABO-incompatible transplantation was performed. Because of the ABO incompatibility, the recipient received rituximab followed by immunoadsorption in order to reduce the anti-A titers to an acceptable level. Nine hours after transplantation, recurrent aHUS was evident. This was successfully treated with eculizumab, which has been continued, and graft function remains good (eGFR: 47.6 ml/min per 1.73 m^2^). The other 4 patients received deceased donor kidneys and standard immunosuppression according to the local transplant unit protocol (Table 5) with no specific autoantibody-targeting treatment, and none experienced aHUS recurrence; 3 of 4 patients continue to have good graft function (eGFR: 48.4 ml/min per 1.73 m^2^ in 1 patient and >60 ml/min per 1.73 m^2^ in 2 patients), and 1 of 4 transplants failed after 7.5 years due to severe antibody mediated rejection.

**Table 5:** Outcome of transplantation (*N* = 6)

Patient	Transplant	Age at transplantation	Time after first presentation of aHUS	HLA mismatch	Treatment before transplantation	Induction immunosuppression	Maintenance immunosuppression	Outcome
4^a^	DCD (en bloc)	8 yr, 9 mo	5 yr	Unknown	None	Unknown	Unknown	Primary nonfunction
4^a^	LRT^b^ ABOi	9 yr, 3 mo	5 yr, 6 mo	1:1:1	Rituximab and immunoadsorption^c^	Basiliximab	Tacrolimus, MMF, prednisolone	Immediate recurrence,^d^ eculizumab, creatinine: 72 μmol/l, eGFR: 47.6 ml/min per 1.73 m^2^ (follow-up 9 mo)
5	DBD	12 yr, 6 mo	1 yr, 10 mo	1:1:0	None	Basiliximab	Tacrolimus, azathioprine, prednisolone	Transplant failed after 7 yr 6 mo (severe AMR postpartum)
12	DBD	10 yr	1 yr, 5 mo	Unknown	None	Basiliximab	MMF, prednisolone	Creatinine: 80 μmol/l, eGFR: >60 ml/min per 1.73 m^2^ (follow-up 6 yr 9 mo)
19	DBD	11 yr, 5 mo	1 yr, 5 mo	1:1:0	None	Unknown	Tacrolimus, azathioprine, prednisolone	Creatinine: 109 μmol/l, eGFR: 48.4 ml/min per 1.73 m^2^ (follow-up: 3 yr 11 mo)
22	DCD	9 yr, 9 mo	2 yr	Unknown	None	Basiliximab	Tacrolimus (purine antagonist stopped)	Creatinine: 83 μmol/l, eGFR: >60 ml/min per 1.73 m^2^ (follow-up: 2 yr 5 mo)

ABOi, ABO incompatible; aHUS, atypical hemolytic uremic syndrome; AMR, antibody-mediated rejection; DBD, donor after brain death; DCD, donor after cardiac death; eGFR, estimated glomerular filtration rate; LRT, living related transplant; MMF, mycophenolate mofetil.

eGFR by Schwartz formula (all patients were younger than 18 years of age at last follow-up).

a Concomitant functionally significant *CFI* mutation.

b LRT: living donor underwent genetic screening; no rare genetic variant, 0 copies *CFHR1,* 1 copy *CFHR3*.

c Treatment given because of ABO incompatibility: rituximab 1 month before transplant, and immunoadsorption ×4 (anti–A titers reduced from 1:128 to 1:8).

d Recurrence 9 hours after transplantation. Successfully treated with eculizumab.

## Discussion

In this report, we describe the phenotypic, immunologic, and genetic characteristics of children younger than 16 years of age presenting in the United Kingdom and Ireland since 2000 with FH autoantibody–associated aHUS and evaluate treatment and outcomes.

The median age at presentation was 8 years, and there was a male predominance, which concurs with other studies.^6^, ^15^, ^22^ Similar to other European cohorts (Supplementary Table S4),^15^, ^22^, ^23^ we found that gastrointestinal prodromal symptoms were commonly reported, which may lead to difficulty differentiating from Shiga toxin–associated HUS with a consequent delay in appropriate treatment.

The clinical presentation of the patients in an Indian cohort reported by Sinha *et al.*^6^ is different, with diarrhea reported in only 9%; the other striking difference in this cohort is the higher incidence of anti-FH autoantibodies (56% compared with 13% in this study, 5%–25% in European cohorts and 29.4% in a Korean cohort)^5^, ^6^, ^24^ (Supplementary Table S4). It may be that environmental factors, for example, endemic infectious disease, or as yet unidentified genetic factors are having a significant influence on disease manifestation.

In our study, 4 of 17 patients had a low C3 level, which is a lower proportion than in other cohorts (Supplementary Table S4),^6^, ^15^, ^19^, ^22^, ^24^, ^25^ and we did not find that C3 level correlated with prognosis, in contrast to Dragon-Durey *et al.*^22^ and Sinha *et al.*^6^ Five of 17 patients had a low C4 level despite FH being a complement alternative pathway regulator; other groups have also reported low C4 levels in a small proportion of patients.^9^, ^15^, ^19^, ^25^ It is interesting that all patients with low C4 also had circulating FH/autoantibody immune complexes and that there was a correlation between the immune complexes titer and C4 level (Supplementary Figure S3). This could suggest that the complexes themselves are activating the classic pathway of complement.

In 10 patients, anti-FH autoantibody titer measurements at multiple time points were available (Figure 3). In 5 patients who had transplants and therefore receive long-term immunosuppression, the titers have decreased. It is interesting that in 3 patients who have been treated with eculizumab for several years and remain well, the autoantibody titers have increased compared with the titers at presentation. It is also interesting that 2 patients still have positive autoantibody titers 9 and 12 years after presentation, yet only 1 experienced a relapse (single relapse at 88 days), and both remain well despite not receiving any specific long-term treatment.

Low FH levels were reported previously in 22% to 29%^22^, ^25^ (1 study included 2 patients with a *CFH* rare genetic variant).^25^ In our study, 2 of 17 patients had a low FH level despite having no rare genetic variants in *CFH*; both patients had circulating immune complexes, and we assume the low FH level is secondary to this complexing.

We identified rare genetic variants in genes encoding complement components or complement regulators in 7 of 17 patients, and at least some are functionally significant. In comparison, Dragon-Durey *et al.*^22^ did not identify any rare genetic variants in 26 patients, but other studies have identified rare genetic variants in 4% to 33% of patients with FH autoantibodies^15^, ^23^, ^25^, ^26^ (Supplementary Table S4); genetic analysis data have not been reported for the Indian cohort.^6^ This has crucial implications for management: if treatment is focused exclusively on antibody removal and reduction without considering that a concomitant functionally significant rare genetic variant may also be contributing to the disease, then it may be ineffective. We therefore recommend genetic analysis of all patients with FH autoantibodies.

In agreement with other studies,^6^, ^12^, ^14^, ^15^, ^17^, ^19^, ^22^, ^23^, ^24^, ^25^ we found that the majority of patients with anti-FH autoantibody aHUS have a homozygous deletion of *CFHR1*; Nozal *et al.*^10^ previously demonstrated that the presence of deficiency of FH-related protein 1 is associated with differences in antibody binding, and this was also the case in our study. Whereas most patients with a homozygous deletion of *CFHR1* had antibodies that predominantly bound to the C-terminal of FH, in 2 of the 3 patients with 2 copies of *CFHR1*, the antibodies bound predominantly to the N-terminal of FH.

Historically, anti-FH autoantibody–associated aHUS was associated with a high rate of ERF (27%–63%).^6^, ^19^, ^22^, ^25^ Consistent with this, in our cohort, ERF developed in 6 of 17 patients, although this may reflect late presentation rather than unsuccessful treatment. Lee *et al.*^24^ reported the only study in which ERF did not develop in any patients in a Korean cohort of 15 patients. In our study, 8 of 17 patients required dialysis within 1 week of presentation, but 6 of 8 of these fully recovered renal function, so dialysis requirement at presentation did not predict poor renal outcome.

There are no clinical trials investigating treatment of anti-FH autoantibody aHUS, and evidence comes only from retrospective cohorts. In the 2 patients in our cohort who received supportive treatment only, ERF developed in both, and in a French cohort, only 1 of 6 patients managed supportively had no renal sequelae.^22^ In the United Kingdom and Ireland, of the 11 patients treated with PEX at initial presentation, ERF developed in 4, a rate in keeping with other reports.^22^

High rates of late relapse have been observed in those patients successfully treated with PEX^22^ following cessation, and many centers have recommended additional immunosuppression. It has been demonstrated that this results in significant reductions in autoantibody titer.^6^, ^27^, ^28^ The United Kingdom and Ireland are unusual in not adopting immunosuppressive regimens after PEX due to concerns about the high incidence of concomitant rare genetic variants in complement genes and the potential side effects of cytotoxic therapy in a pediatric population, which include infectious complications, gonadal toxicity, and long-term risk of malignancy.^29^ Five of the 7 children who recovered renal function after PEX subsequently experienced relapse, but careful monitoring with reintroduction of treatment resulted in recovery of renal function in all cases.

Dragon-Durey *et al.*^22^ reported sustained remission and normal renal function in 3 patients treated with PEX plus cyclophosphamide or mycophenolate mofetil, and Sana *et al.*^28^ also reported good outcomes in 4 patients treated with PEX plus corticosteroids plus pulsed cyclophosphamide; in this latter study, no treatment-related adverse events were observed. Guan *et al.*^30^ reported the successful treatment of 4 patients in a Chinese cohort with steroids and mycophenolate mofetil. Sinha *et al.*^6^ reported the outcome of PEX plus immunosuppression in an Indian cohort of 138 children who received induction therapy with cyclophosphamide (*n* = 49) or rituximab (*n* = 18) and maintenance therapy comprising corticosteroid with or without mycophenolate mofetil or azathioprine (*n* = 47). ERF developed in 32.8%, the relapse rate was low (11.5%), and the mortality rate was 16.4%, with the deaths attributed to complications of renal failure or sepsis.^6^ It is noteworthy that in this cohort, 4 patients experienced relapse despite maintenance immunosuppression.^6^

In a recent review, Dragon-Durey *et al.*^5^ summarized outcomes according to treatment strategy for 243 patients in published studies; for patients treated with PEX and immunosuppression compared with those treated with PEX alone, the relapse rate was 10.7% compared with 28.7%, and the development of chronic kidney disease stage 4/5 or death was 14.3% compared with 35.6%.

Successful use of the terminal complement inhibitor eculizumab in anti-FH autoantibody–associated aHUS has been reported in a small number of patients in trials^31^, ^32^ and case reports.^33^, ^34^, ^35^, ^36^, ^37^ In our study, 6 patients were treated with and continue on eculizumab: 4 of 6 were started on eculizumab at the first presentation, 1 of 6 at relapse, and 1 of 6 following early recurrence after transplantation, with sustained remission observed in all; 3 patients have stable chronic kidney disease stage 3A, and 3 patients have an eGFR >60 ml/min per 1.73 m^2^. There have been no deaths in our cohort, and no adverse events associated with eculizumab. All patients started on eculizumab remain on continuous treatment regardless of autoantibody titer; this is consistent with our current practice in the United Kingdom for all patients with aHUS. Systematic assessment of eculizumab withdrawal in the context of a clinical trial is required, and a prospective trial is due to start recruiting in the United Kingdom.

There is currently insufficient evidence for consensus guidelines on the management of renal transplantation specifically in individuals with anti-FH autoantibody–associated aHUS,^20^ and practice is guided by retrospective analysis of outcomes in published cases, of which there are only 27 transplants in 21 patients,^5^ including 3 living related transplants.^38^, ^39^ There has been considerable variation in peritransplant management strategy, including PEX, rituximab, i.v. IgG, and eculizumab, as well as no specific autoantibody-targeting therapy.^5^, ^40^, ^41^ In their review, Dragon-Durey *et al.*^5^ report that for 9 grafts with no specific management, 5 were lost because of disease recurrence, and for 12 grafts with specific management (including PEX, rituximab, i.v. IgG, and eculizumab), recurrence was seen in 1 (and treated successfully with PEX and eculizumab^42^), and no grafts were lost.

We report the outcomes of renal transplantation in 5 patients with anti-FH autoantibody–associated aHUS (Table 5). None of the patients received specific FH autoantibody–targeting treatment before transplantation, although 1 patient received rituximab and immunoadsorption because of ABO incompatibility, and that patient was the only one in whom aHUS recurrence occurred, although this may have been a consequence of the functionally significant *CFI* mutation rather than the FH autoantibodies.

There is no clearly effective strategy to accurately predict the risk of recurrence following transplantation, and our current practice takes a pragmatic approach: if the anti-FH autoantibody titer is above the positive threshold or if a concomitant functionally significant rare genetic variant is identified, then we recommend prophylactic eculizumab.

The limitations of this study relate to the retrospective and observational methodology, and the significant technological advancement (mainly, the availability of eculizumab) that occurred over the study period. The small sample size is the major factor that limits the strength of the conclusions that can be drawn.

In summary, we report that all patients treated with eculizumab at initial presentation recovered renal function, whereas PEX resulted in only a 64% recovery rate, with supportive treatment universally ineffective. In much of Europe and India, immunosuppression has been added with the aim of preventing disease relapse. This practice was not adopted in the United Kingdom due to concerns over infectious complications and the high incidence of concomitant rare genetic variants. In the absence of immunosuppressive therapy, in those who responded to PEX, the relapse rate was high (71%); however, close monitoring and reintroduction of treatment resulted in recovery of renal function in all cases. In comparison, no patient who continued on eculizumab had recurrent disease. Our current practice is to initiate eculizumab therapy rather than plasma exchange with or without supplemental immunosuppression for FH autoantibody–mediated aHUS, and based on this retrospective analysis, we see no suggestion that this is inferior, albeit the strength of our conclusions is limited by the small sample size.

## Methods

### Patients

All children younger than 16 years of age from the United Kingdom and Ireland presenting since 2000 with aHUS and anti-FH autoantibodies at a titer above the international consensus positive threshold of 100 relative units^21^ were identified from the UK National Renal Complement Therapeutic Centre. A total of 175 children younger than 16 years of age from the United Kingdom and Ireland were tested for anti-FH autoantibodies. Twenty-two children (13%) were positive. Of these, 17 were included in this study, and 5 were excluded (details in Supplementary Methods). Serum samples taken at the time of presentation were available for all included patients. Immunologic and genetic data from 6 of these patients were reported previously by our group^9^; the patient identification numbers used here for these patients (patients 2, 4, 5, 6, 10, and 12) are consistent with those used in the previous publication to allow for direct comparison. Seven patients included previously were excluded from this study because they were not from the United Kingdom and Ireland (patients 1, 7, 8, 9, 11, and 13) or the anti-FH autoantibody titer was found to be below the international consensus positive threshold (patient 3). Data for patients 12^43^ and 16 and 21^44^ were published previously. The details of the eGFR calculations are included in Supplementary Methods. Relapse was defined as disease recurrence >1 month after initial presentation and >15 days after disease remission.

The study was approved by the Northern and Yorkshire Multi-Centre Research Ethics Committee, and informed consent obtained in accordance with the Declaration of Helsinki.

### FH autoantibody assay

The consensus assay was performed as previously described,^21^ and the FH autoantibody was confirmed by Western blotting (details provided in Supplementary Methods).

### Complement assays

C3 and C4 levels were measured by rate nephelometry (Beckman Coulter Array 360, Beckman Coulter; High Wycombe, United Kingdom). FH and factor I levels were measured by radioimmunodiffusion (Binding Site, Birmingham, UK). The normal ranges were C3 (0.68–1.38 g/l), C4 (0.18–0.60 g/l), FH (0.35–0.59 g/l), and factor I (38–58 mg/l). CD46 was measured as previously described.^45^

### Genetic analysis

Mutation screening of *CFH*,^46^ *CFI*,^47^ *CFB*,^48^ *CD46*,^49^ *C3*,^50^ and *DGKE*^51^ was undertaken using Sanger sequencing, as previously described. Screening for chromosomal rearrangements affecting *CFH*, *CFHR1*, *CFHR2*, *CFHR3*, *CFHR4*, *CFHR5*, *CFI*, and *CD46* was undertaken using multiplex ligation-dependent probe amplification, as previously described.^52^, ^53^

### Statistical analyses

Patient characteristics were examined using descriptive statistics for continuous variables (mean, median) and categorical variables (number, %). Laboratory data are presented as mean (range). Data were analyzed by a 2-tailed Student *t* test. A *P* value <0.05 was considered to be statistically significant. Renal survival was examined using Kaplan-Meier analysis.

## Disclosure

SJ, RDG, THJG, EW, NW, and DK have received honoraria for consultancy work from Alexion Pharmaceuticals. SJ is a member of the Alexion Global aHUS Registry Scientific Advisory Board. AA, RDG, and SH have received support from Alexion Pharmaceuticals to attend conferences. KJM is a scientific consultant for Gemini Therapeutics. DK is a director of and scientific advisor to Gyroscope Therapeutics. All the other authors declared no competing interests.

## Supplementary Material

Supplementary Methods

Figure S1 Serum creatinine and platelet values at presentation.

Figure 2 Initial complement antigenic levels. (**A**) C3. Normal in 76% of patients. (**B**) C4. Normal in 71% of patients. (**C**) Factor I. Normal in all patients. The dashed lines represent the lower limit of the normal ranges.

Figure S3 C4 level and factor H (FH) autoantibody circulating immune complexes. RU, relative units.

Figure S4 Outcome according to dialysis requirement at presentation; 75% of patients who required dialysis within 1 week of presentation fully recovered renal function. *Defined as dialysis within the first week of presentation. **Defined as recurrence >1 month after presentation and >15 days after disease remission. †Management: supportive: 1, PEX: 1. ‡Management: PEX: 4, eculizumab: 2. ERF, established renal failure; PEX, plasma exchange.

Figure S5 Kaplan-Meier survival curve showing probability of renal survival. Established renal failure developed in 35% of patients, and in all cases, this occurred at the first presentation of atypical hemolytic uremic syndrome. There were no deaths.

Table S1 Autoantibody reactivity with short factor H fragments.

Table S2 Initial titers of factor H autoantibody (aFH), circulating immune complexes (CiC), and autoantibody reactivity with factor H–related proteins 1 to 5 (positive threshold >100 relative units).

Table S3 Treatment modality and resultant renal function.

Table S4 Comparison with other cohorts.
