# Supplementary material for: Factor H autoantibody is associated with atypical hemolytic uremic syndrome in children in the United Kingdom and Ireland
Source: Kidney Int. 2017 Nov;92(5):1261–71. doi: 10.1016/j.kint.2017.04.028 (PMC5652378; doi:10.1016/j.kint.2017.04.028)
Supplement: Supplementary Methods [file mmc1.pdf]

Supplemental Methods

Details of excluded patients

5 of 22 children from the UK and Ireland presenting with aHUS and anti-FH autoantibodies at a titre above the international consensus positive threshold of 100 RU were excluded from this study because: 1 child had Shiga toxin associated HUS and the low titre autoantibody was not felt to be significant; 1 individual presented aged 7 years with aHUS in 1992, but did not have serum tested for autoantibodies until 22 years later (the clinical course was of multiple relapses in childhood, treated with plasma exchange (PEX) and intravenous immunoglobulin (IVIG), and peritoneal dialysis for year before recovery of renal function – which has been stable since); 1 individual had serum tested for autoantibodies 10 years after presenting with aHUS; in 2 individuals the initial assay was positive but no samples were available to confirm positivity using the international consensus assay.

eGFR calculation

The estimated glomerular filtration rate (eGFR) was calculated: for children (<18 years), the Schwartz formula was used: eGFR (mL/min/1.73 m^2) = [0.55 x height (centimetres) x K (constant)] / serum creatinine (μmol/L) x 0.0113 (correction factor for mg/dL); in first year of life, for pre-term babies K=0.33 and for full-term infants K=0.45; for infants and children between ages of 1 and 12 years, K=0.55; adolescent boys, K=0.7. For adults the abbreviated MDRD equation was used: 186 x $(\text{Creat} / 88.4)^{-1.154}$ x $(\text{Age})^{-0.203}$ x (0.742 if female) x (1.210 if black).

Factor H autoantibody assay

The consensus assay was performed as previously described[1]. A 96 well Maxisorb^{tm} ELISA plate (Nunc) was coated with 50μl/well of purified factor H (CompTech, Tyler, Texas, USA) at 5μg/ml or molar equivalents of factor H fragments (short consensus repeats [SCRs] 1-7 (generated in house) 8-15, 19-20)[2, 3] or factor H–related protein 1 fragment (SCRs 4-5)[4] in Dulbecco's PBS (137 mM NaCl, 2.7 mM KCl, 8.1 mM Na_2HPO_4, 1.76 mM KH_2PO_4, pH 7.4) and incubated overnight at 4°C. Alternatively, for detection of circulating immune complexes (CIC) plates were coated with OX24 (2μg/ml) in 0.1M carbonate buffer, pH 9.6. Plates were washed once with PBS, then blocked with 200μl PBS/0.1% Tween (PBS-T) per well for 1hr at room temperature (for CIC, plates were blocked with 1% BSA/PBS-T). A second (replicate) plate was incubated with block only. Plates were washed 3 times with PBS/0.1% Tween. Individual test samples were diluted then 1/50 in PBS-T and 50 μl applied in triplicate to each plate, including a positive, negative and 'no serum' control. A standard curve was established using positive sample applied in doubling dilution from 1/25 to 1/3200. Samples and controls were incubated for exactly 1hr at room temperature followed by 3 washes with PBS-T. A 1/20,000 dilution of HRP conjugated goat anti-human IgG (Stratech Scientific Ltd, UK) in PBS-T (50μl/well) was then applied for exactly 1 hour. Both test and control plates were washed 3 times with PBS-T, 50μl/well of TMB substrate was applied (readymade,

Leico Technologies) and plates developed for 5-10min. The reaction was stopped using 50µl/well 10% H_2SO_4 and OD450nm measurements taken using an EL-800 plate reader (Biotek, UK). The readings from the 'block only' plate were then subtracted from the factor H coated plate. An ELISA was considered valid when the range between positive and negative control was greater than 1.0 (OD450). Readings from the background subtracted positive control standard curve were assigned 4000 relative units (RU) for a 1/25 dilution of Newcastle positive serum. These were plotted using PrismGraph 3 software, which allowed automatic interpolation of sample RU from a curve fit based on four-parameter logistic non-linear regression. Samples were analysed on three individual experiments and the mean of these used in the figures herein.

Western blotting confirmation of factor H autoantibody.

Purified complement factor H (Comptech) was diluted in solubilizing buffer (non-reducing) and loaded onto a 10% sodium dodecyl sulfate–polyacrylamide gel electrophoresis (SDS-PAGE) preparative gel. After transfer to nitrocellulose and blocking as described previously, the nitrocellulose was cut into 0.5- to 1-cm wide strips. These strips were then incubated with individual sera samples (1/100 in 5% dried milk/PBS) for 1 to 2 hours at room temperature. After washing as described previously, bound autoantibody was detected by the use of goat anti-human IgG-HRP incubated for 1 to 2 hours at room temperature. Blots were then washed twice with PBS/0.01% Tween 20 and with PBS only. All blots were developed by the use of an enhanced chemiluminescence (ECL) substrate (Pierce) according to the manufacturer's specifications.

1. Watson R, Lindner S, Bordereau P*, et al.* Standardisation of the factor H autoantibody assay. *Immunobiology* 2014; **219:** 9-16.

2. Hocking HG, Herbert AP, Kavanagh D*, et al.* Structure of the N-terminal region of complement factor H and conformational implications of disease-linked sequence variations. *The Journal of biological chemistry* 2008; **283:** 9475-9487.

3. Schmidt CQ, Herbert AP, Kavanagh D*, et al.* A new map of glycosaminoglycan and C3b binding sites on factor H. *Journal of immunology* 2008; **181:** 2610-2619.

4. Ferreira VP, Herbert AP, Cortes C*, et al.* The binding of factor H to a complex of physiological polyanions and C3b on cells is impaired in atypical hemolytic uremic syndrome. *Journal of immunology* 2009; **182:** 7009-7018.
