# Supplementary figures and images for: Factor H autoantibody is associated with atypical hemolytic uremic syndrome in children in the United Kingdom and Ireland
Source: Kidney Int. 2017 Nov;92(5):1261–71. doi: 10.1016/j.kint.2017.04.028 (PMC5652378; doi:10.1016/j.kint.2017.04.028)

**Supplemental Figure 1: Serum creatinine and platelet values at presentation**

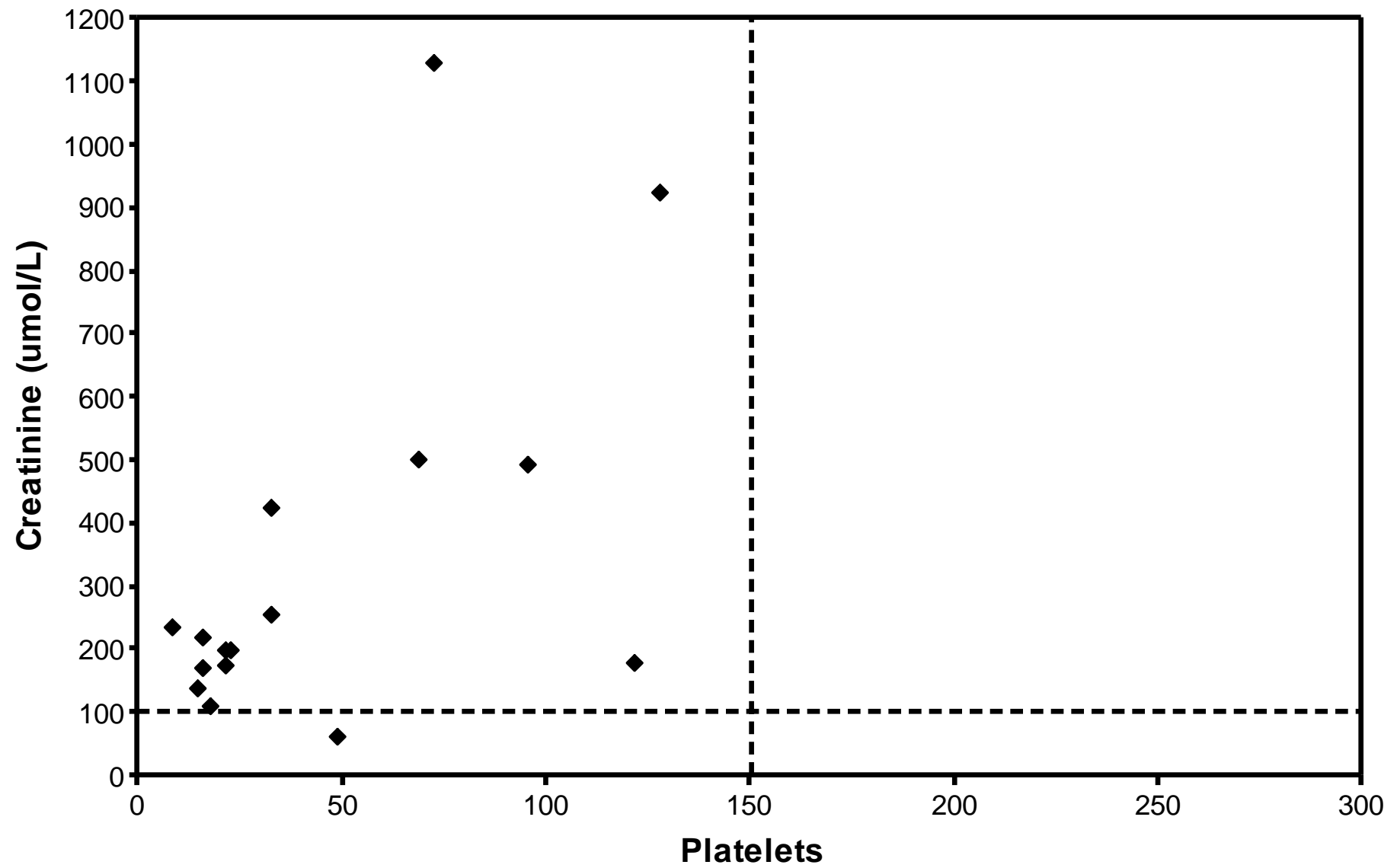

Supplement: Figure S1 — Serum creatinine and platelet values at presentation. [file mmc2.pdf]
