# Supplementary material for: Factor H autoantibody is associated with atypical hemolytic uremic syndrome in children in the United Kingdom and Ireland
Source: Kidney Int. 2017 Nov;92(5):1261–71. doi: 10.1016/j.kint.2017.04.028 (PMC5652378; doi:10.1016/j.kint.2017.04.028)

Supplemental Figure 2: Initial complement antigenic levels. A. C3. Normal in 76% of patients. **B**. C4. Normal in 71% of patients. **C**. Factor I. Normal in all patients.
The dashed lines represent the lower limit of the normal ranges.

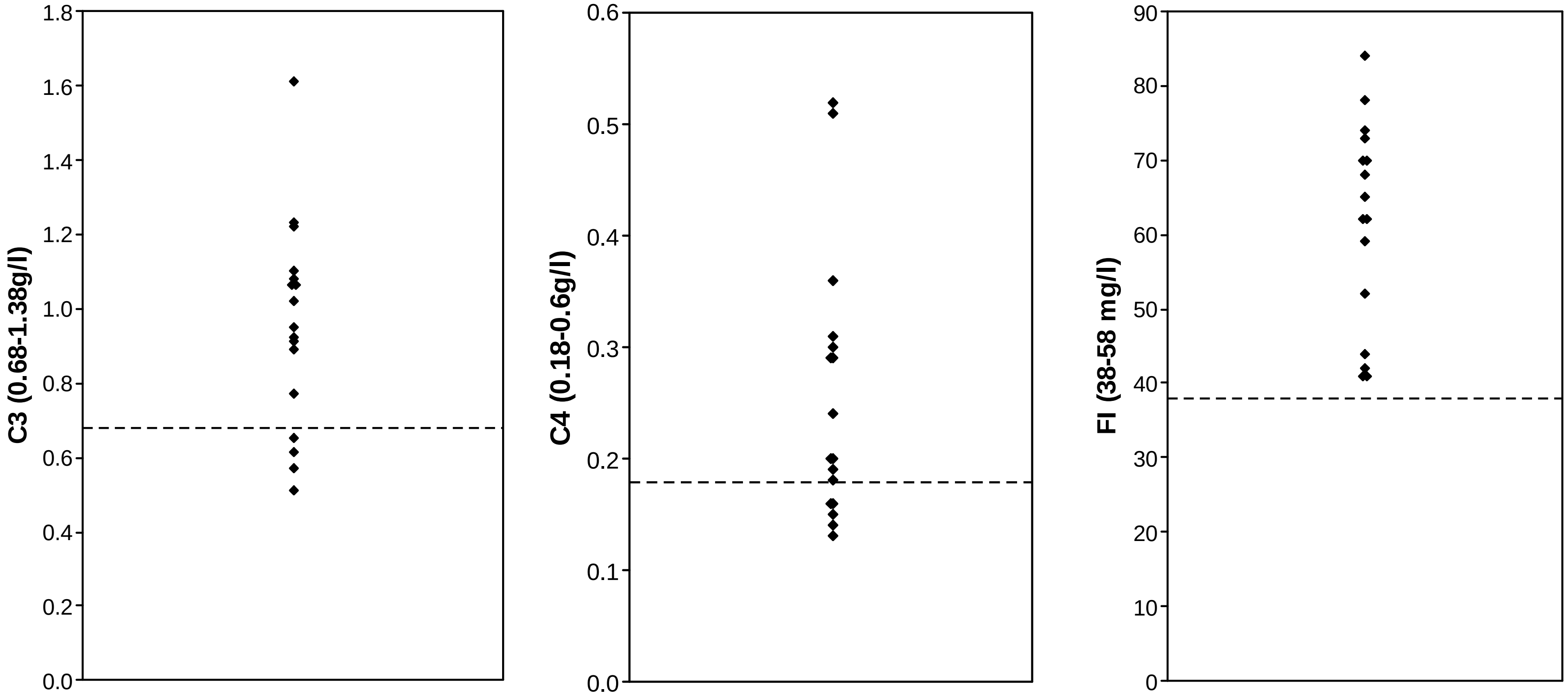

Supplement: Figure 2 — Initial complement antigenic levels. (A) C3. Normal in 76% of patients. (B) C4. Normal in 71% of patients. (C) Factor I. Normal in all patients. The dashed lines represent the lower limit of the normal ranges. [file mmc3.pdf]
