# Supplementary material for: Factor H autoantibody is associated with atypical hemolytic uremic syndrome in children in the United Kingdom and Ireland
Source: Kidney Int. 2017 Nov;92(5):1261–71. doi: 10.1016/j.kint.2017.04.028 (PMC5652378; doi:10.1016/j.kint.2017.04.028)

Supplemental Figure 3: C4 level and FH/autoantibody circulating immune complexes

Abbreviations: RU, relative units

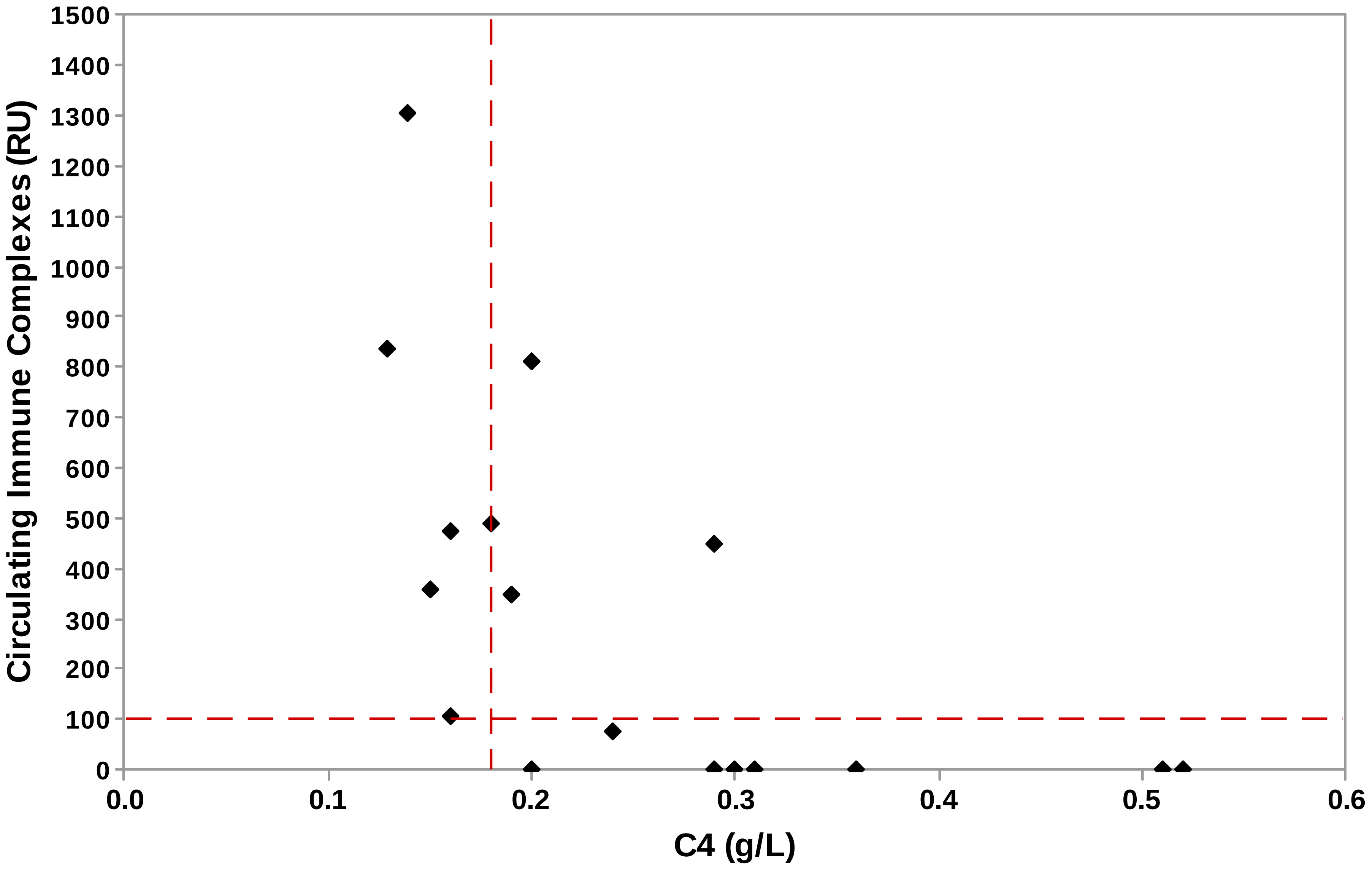

Supplement: Figure S3 — C4 level and factor H (FH) autoantibody circulating immune complexes. RU, relative units. [file mmc4.pdf]
