# Supplementary material for: Factor H autoantibody is associated with atypical hemolytic uremic syndrome in children in the United Kingdom and Ireland
Source: Kidney Int. 2017 Nov;92(5):1261–71. doi: 10.1016/j.kint.2017.04.028 (PMC5652378; doi:10.1016/j.kint.2017.04.028)

Supplemental Figure 4: Outcome according to dialysis requirement at presentation.

75% of patients who required dialysis within one week of presentation fully recovered renal function.

*Defined as dialysis within the first week of presentation

**Defined as recurrence >1 month after presentation and >15 days after disease remission

† Management: supportive:1, PEX:1

‡ Management: PEX:4, eculizumab: 2

Abbreviations: ERF, established renal failure; PEX, plasma exchange.

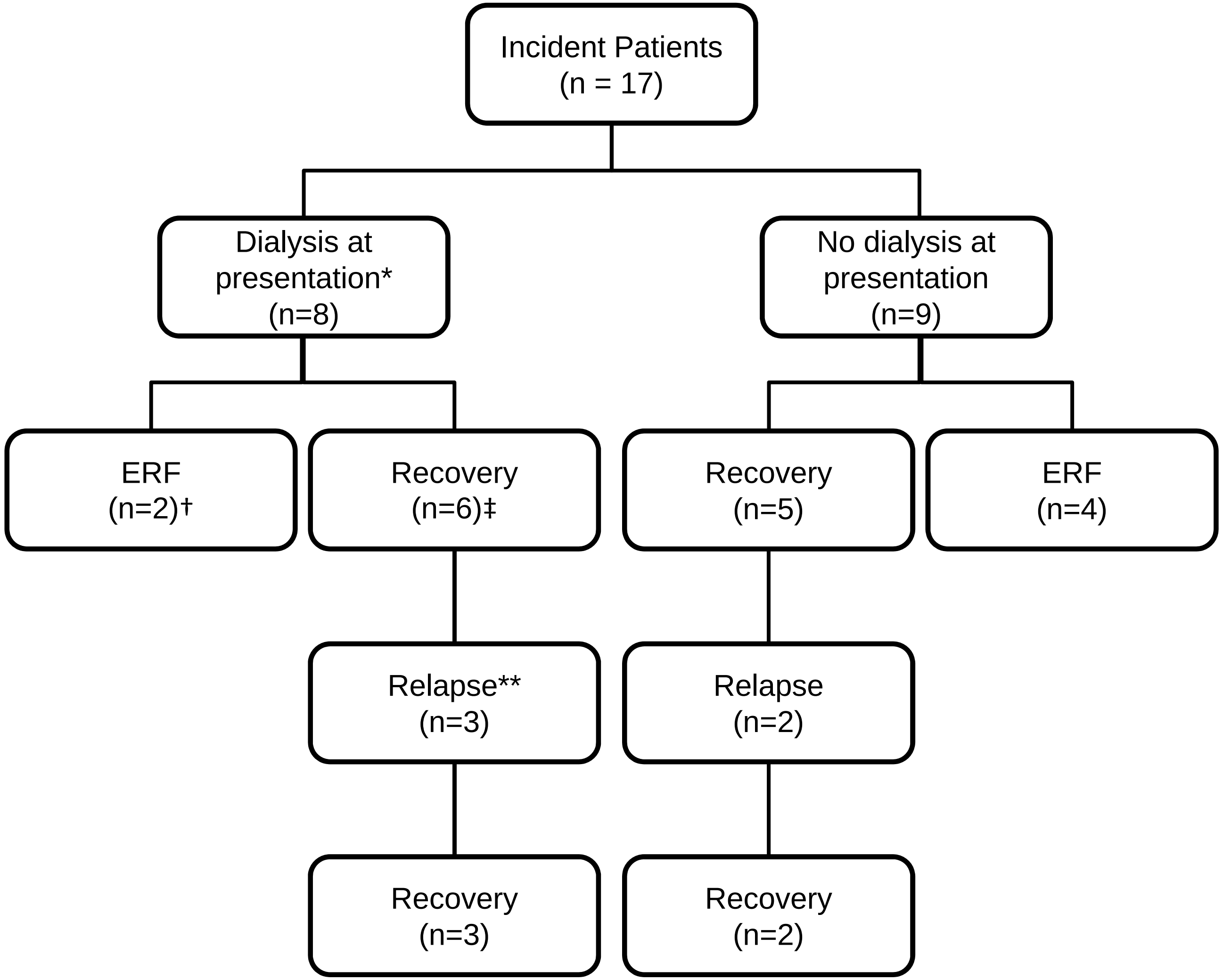

Supplement: Figure S4 — Outcome according to dialysis requirement at presentation; 75% of patients who required dialysis within 1 week of presentation fully recovered renal function. *Defined as dialysis within the first week of presentation. **Defined as recurrence >1 month after presentation and >15 days after disease remission. †Management: supportive: 1, PEX: 1. ‡Management: PEX: 4, eculizumab: 2. ERF, established renal failure; PEX, plasma exchange. [file mmc5.pdf]
