# Supplementary material for: Factor H autoantibody is associated with atypical hemolytic uremic syndrome in children in the United Kingdom and Ireland
Source: Kidney Int. 2017 Nov;92(5):1261–71. doi: 10.1016/j.kint.2017.04.028 (PMC5652378; doi:10.1016/j.kint.2017.04.028)
Supplement: Figure S5 — Kaplan-Meier survival curve showing probability of renal survival. Established renal failure developed in 35% of patients, and in all cases, this occurred at the first presentation of atypical hemolytic uremic syndrome. There were no deaths. [file mmc6.pdf]

Supplemental Figure 5: Kaplan Meier survival curve showing probability of renal survival

35% of patients developed ERF, and in all cases this occurred at the first presentation of aHUS. There were no deaths.

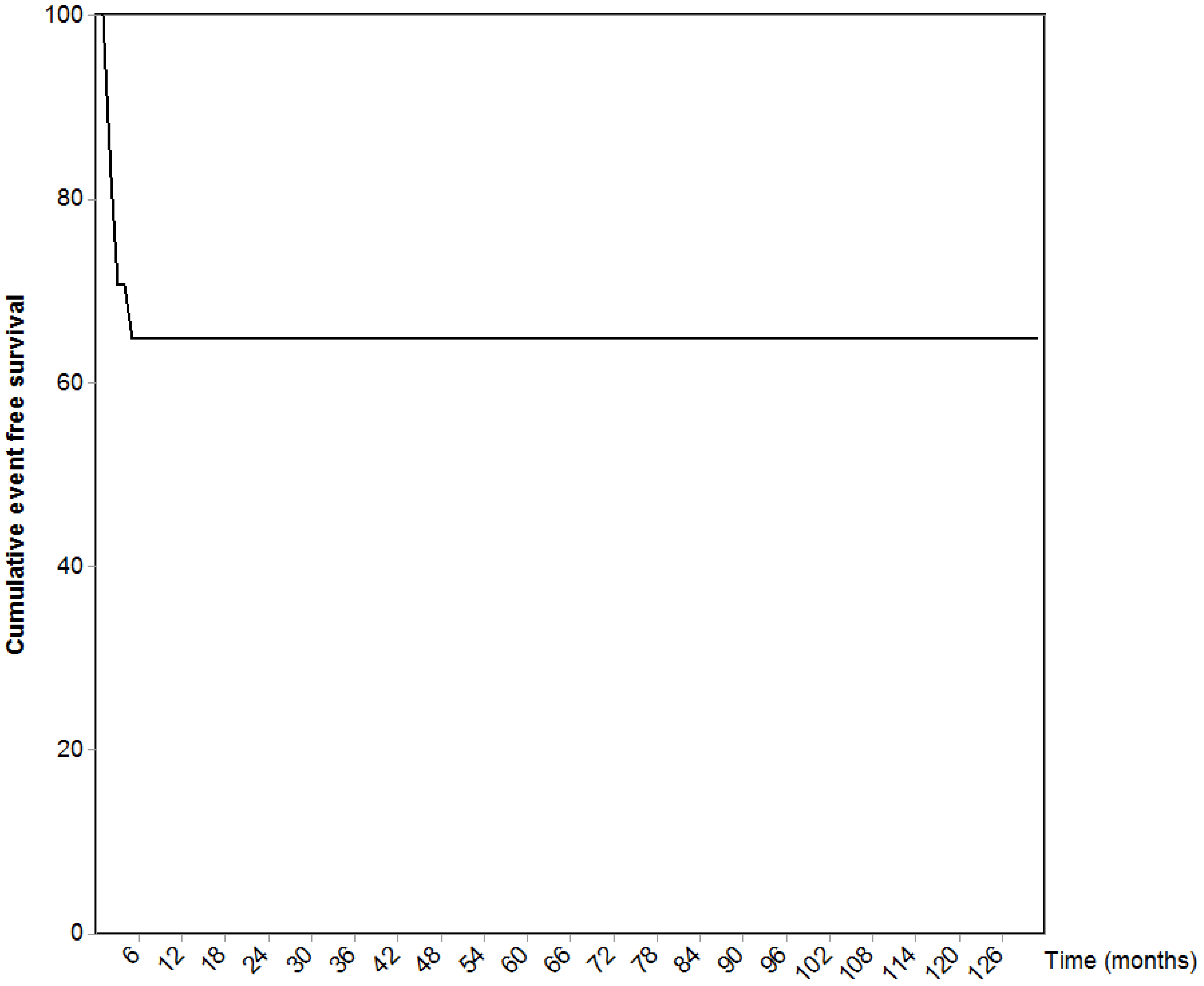

0	6	6	6	6	6	6	6	6	6	6	6	6	6	6	6	6	6	6	6	6	6	Endpoint
17	11	8	6	6	6	6	5	5	4	4	4	4	4	3	3	3	3	3	3	2	1	N at risk
