# Supplementary material for: Factor H autoantibody is associated with atypical hemolytic uremic syndrome in children in the United Kingdom and Ireland
Source: Kidney Int. 2017 Nov;92(5):1261–71. doi: 10.1016/j.kint.2017.04.028 (PMC5652378; doi:10.1016/j.kint.2017.04.028)
Supplement: Table S1 — Autoantibody reactivity with short factor H fragments. [file mmc7.pdf]

Supplemental Table 1: Autoantibody reactivity with short factor H fragments.

CFHR1 copy number and autoantibody binding to factor H fragments (SCRs 1-7, 8-15, 16-18 and 19-20) (positive threshold >100RU).

Abbreviations: SCR = Short Consensus Repeat.

Patient	*CFHR1* copy number	SCRs 1-7	SCRs 8-15	SCRs 16-18	SCRs 19-20
**2**	0	130	0	24	613
**4**	0	7	0	0	2171
**5**	0	235	3	57	144
**6**	2	3126	9	615	0
**10**	0	0	0	0	1570
**12**	0	41	450	0	377
**14**	0	33	60	0	2365
**15**	0	7	0	81	2245
**16**	0	0	0	0	239
**17**	0	1	3	0	2370
**18**	0	42	0	0	981
**19**	2	5	0	0	410
**20**	0	46	0	0	219
**21**	0	496	76	0	2714
**22**	2	327	0	13	1
**23**	0	0	0	0	1050
**24**	0	0	0	104	3243
