# Supplementary material for: Factor H autoantibody is associated with atypical hemolytic uremic syndrome in children in the United Kingdom and Ireland
Source: Kidney Int. 2017 Nov;92(5):1261–71. doi: 10.1016/j.kint.2017.04.028 (PMC5652378; doi:10.1016/j.kint.2017.04.028)
Supplement: Table S2 — Initial titers of factor H autoantibody (aFH), circulating immune complexes (CiC), and autoantibody reactivity with factor H–related proteins 1 to 5 (positive threshold >100 relative units). [file mmc8.pdf]

Supplemental Table 2: Initial titres of factor H autoantibody (aFH), circulating immune complexes (CiC), and autoantibody reactivity with factor H–related proteins 1-5 (positive threshold >100RU)

Abbreviations: aFH, factor H autoantibody; CiC, circulating immune complexes; FHR, factor H related proteins; RU, relative units.

Patient	aFH	CiC	FHR1	FHR2	FHR3	FHR4	FHR5
**2**	630	357	694	74	0	0	0
**4**	1249	0	792	0	0	0	21
**5**	573	812	0	352	147	169	43
**6**	2017	0	0	0	0	0	0
**10**	3432	840	612	0	0	0	0
**12**	812	350	0	0	0	0	0
**14**	722	0	132	0	0	29	3
**15**	4000	450	1380	65	39	0	0
**16**	4000	0	1350	0	0	0	45
**17**	2194	75	600	0	0	0	41
**18**	2130	490	900	22	0	0	0
**19**	2319	0	500	0	0	0	0
**20**	1594	106	0	0	0	0	0
**21**	971	0	700	0	0	0	0
**22**	277	0	0	0	37	30	21
**23**	1350	474	279	0	359	468	341
**24**	3396	1307	2645	0	0	20	0
