# Supplementary material for: Factor H autoantibody is associated with atypical hemolytic uremic syndrome in children in the United Kingdom and Ireland
Source: Kidney Int. 2017 Nov;92(5):1261–71. doi: 10.1016/j.kint.2017.04.028 (PMC5652378; doi:10.1016/j.kint.2017.04.028)
Supplement: Table S3 — Treatment modality and resultant renal function. [file mmc9.pdf]

Supplemental Table 3: Treatment modality and resultant renal function

Patient	Treatment at first presentation	Dialysis (duration if recovery)	ERF	Relapse	Renal Transplant	Subsequent treatment	Duration of follow up (months)	eGFR (mL/min/1.73m^2) at most recent follow up
**2**	FFP, PEX	Yes (5 weeks)	No	Yes	n/a	PEX at relapse	163	>60†
**4**	FFP, PEX, IVIG	Yes	Yes	No	Yes<br>aHUS recurrence	ECU post-transplant	75	47.6*
**5**	PEX	Yes	Yes	No	Yes<br>Graft failure (AMR)		151	ERF
**6**	PEX	Yes	Yes	No	No. Ineligible		130	ERF
**10**	PEX, CS	No	No	Multiple	n/a	Regular PEX, withdrawn 2012	117	>60*
**12**	FFP, PEX, IVIG	Yes	Yes	No	Yes		98	>60*
**14**	FFP, ECU	No	No	No	n/a		7	>60*
**15**	ECU	Yes (5 days)	No	No	n/a		11	>60*
**16**	PEX	Yes (3 days)	No	Yes	n/a	ECU at relapse	81	>53*
**17**	PEX	No	No	No	n/a		108	>60*
**18**	FFP, PEX	No	No	Yes	n/a	PEX at relapse	123	>60*
**19**	SUP	Yes	Yes	No	Yes		64	48.4*
**20**	ECU	No	No	No	n/a		62	>60*
**21**	FFP, PEX	Yes (6 days)	No	Yes	n/a	PEX, RTX at relapse	9	>60†
**22**	SUP	Yes	Yes	No	Yes		84	>60*
**23**	PEX	Yes (5 days)	No	No	n/a		12	>60*
**24**	ECU	Yes (5 weeks)	No	No	n/a		22	52.3*

eGFR: by Schwartz formula for patients <18 years at last follow up (*) and by abbreviated MDRD equation for patients >18 at last follow up (†)

Abbreviations: AMR, antibody mediated rejection; CS, corticosteroids; ECU, eculizumab; eGFR: estimated glomerular filtration rate; ERF, established renal failure; FFP, fresh frozen plasm; IVIG, intravenous immunoglobulin; MMF, mycophenolate mofetil; n/a, not applicable; PEX, plasma exchange; RTX, rituximab; SUP, supportive management.
