# Supplementary material for: Factor H autoantibody is associated with atypical hemolytic uremic syndrome in children in the United Kingdom and Ireland
Source: Kidney Int. 2017 Nov;92(5):1261–71. doi: 10.1016/j.kint.2017.04.028 (PMC5652378; doi:10.1016/j.kint.2017.04.028)
Supplement: Table S4 — Comparison with other cohorts. [file mmc10.pdf]

Supplemental Table 4: Comparison with other cohorts

	**Dragon-Durey *et al.*[22]**	**Sana *et al.*[28]**	**Sinha *et al.*[6]**	**Lee *et al.*[24]**	**Geerdink *et al.*[23]**	**Noris *et al.*[25]**	**Abarrategui-Garrido *et al.*[19]**	**Hofer *et al.*[15]**	**Moore *et al.*[9]**	**UK and Ireland**
**# Patients**	45 (38 children)	4 children	138 children	15 children	6 children	8 (6 children)	7 children	25 children	13 children	17 children
**Age (years)**	Median 8.5 (children only)	Mean 6	Mean 8.4	Mean 8	1-7 years at onset in 83.3%	N/A	Mean 5.2	Mean 7.9	Median 8	Median 8
**% Male**	66% (children only)	N/A	72.5%	27%	50%	50%	N/A	N/A	46%	65%
**Gastrointestinal symptoms**	84% (diarrhoea 53%)	25%	Diarrhoea 9.4%	Diarrhoea 13%	83%	14.3%	N/A	87%	N/A	75% (diarrhoea 50%)
**Extra-renal manifestations**	50% hepatitis<br>23% pancreatitis<br>23.5% seizures	N/A	57.3% hepatic<br>40.6% seizures	7% hepatitis<br>7% pancreatitis<br>7% CNS	N/A	14.3% CNS	N/A	11% CNS<br>58% other	N/A	19% seizures<br>13% hepatitis<br>6% pancreatitis
**Concomitant rare genetic variants**	0/26	0	N/A	0/15	2/6 (33%): *CFI, C3*	2/8 (25%): *CFH* x2	0/7	1/8 (12.5%): *CFI*	5/13 (38%): *CFH, CFI, CD46, C3* x2	7/17 (41%): *CFH, CFI* x4, *CD46, C3*
**Complement analysis** **Low C3**	58%	50%	62%	67%	N/A	43%	57%	41%	27%	24%
**Low C4**	0	N/A	N/A	N/A	N/A	14%	14%	15%	27%	29%
**Low FH**	22%	N/A	N/A	N/A	N/A	29%	0	N/A	9%	12%
**Treatment and evolution of first episode**										
Supportive	6 (13% of total): ERF 1 (17%); relapses 2 (33%); TR 1 (17%); CKD 1 (17%)	0	N/A	N/A	2 (33%)	N/A	N/A	N/A	10 (77%)	2 (12% of total): ERF 2 (100%)
PI	6 (13% of total): relapses 5 (83%), TR 1 (17%)	0	N/A	91%	N/A	N/A	N/A	N/A	N/A	n/a
PEX	15 (33% of total): ERF 1 (7%); death 1 (7%); CKD 3 (20%); TR 3 (20%); relapses 6 (40%)	0	105 (76%) in total	69%	N/A	N/A	N/A	N/A	3 (23%)	11 (65% of total): ERF 4 (36%); relapses 5 (45%)
PEX or PI	N/A	0	N/A	N/A	4/6 (67%) (chronic in 3)	7/8 (87.5%)	5/7 (71%)	18/19 (95%)	N/A	n/a
PEX + Corticosteroids	0	0	N/A	N/A	N/A	3/8 (37.5%): remission in 2/3 (67%)	1	N/A	N/A	1
PEX + Immunosuppression	3 (6.7% of total)	4 (100%)	87 (63%) IS	7 (54%) steroids +/- IS	0	3/8 (37.5%)	2 (29%)	0	0	0
Azathioprine	0	0	N/A	N/A	n/a	0	1	n/a	n/a	n/a
Steroids + cyclophosphamide	2: no renal sequelae	4 (at relapse in patient treated with rituximab)	49	N/A	n/a	0	0	n/a	n/a	n/a
Steroids + rituximab	0	(rituximab initially in 1)	18	N/A	n/a	0	0	n/a	n/a	n/a
Steroids + MMF	1: no renal sequelae	0	N/A	N/A	n/a	0	0	n/a	n/a	n/a
Eculizumab	0	0	0	0	0	0	0	0	0	4 (24% of total): 100% sustained remission
Maintenance Immunosuppression	N/A	n/a	47 (34%): steroids alone 21; plus azathioprine 8; plus MMF 18	N/A	n/a	N/A	N/A	n/a	n/a	0
**Long term outcomes**										
Sustained remission	N/A<br>(25% no renal sequelae)	100% after cyclophosphamide	N/A	62% no sequelae	0	12% complete remission	N/A	N/A	N/A	n/a
Relapse	58%	25% (following rituximab in 1)	14 (11.5%);<br>(4 whilst on IS)	31%	50%	37.5%	N/A	N/A	23%	29% overall
ERF	27%	0	32.8%	0	0	63%	43%	N/A	46%	35% overall
CKD (not including ERF)	39%	0	N/A	15%	N/A	N/A	N/A	N/A	N/A	CKD stage 3: 1 (6%)
Death	4 (8.9%)	0	20 (16.4%)	0	1 (16.7%)	0	1 (14.3%)	N/A	0	0
	Deaths: unknown; sudden death post dialysis; pulmonary hypertension; cardiac insufficiency	n/a	Deaths: complications of renal failure 16; sepsis 4	n/a	N/A	n/a	Death: myocarditis	N/A	n/a	n/a
**Outcome of transplantation**										
Recurrence	3 without specific management	n/a	0	n/a	0	1	0	N/A	0	1 early recurrence (*CFI* mutation) treated with Eculizumab
No recurrence	3 with specific management	n/a	3 with specific management (PEX/IVIG/rituximab)	n/a	0	0	2	N/A	3	4 without specific management (1 graft loss rejection)

Abbreviations: CKD, chronic kidney disease; ERF, established renal failure; FH, factor H; IS, immunosuppression; IVIG, intravenous immunoglobulin; N/A, not available; n/a, not applicable; PEX, plasma exchange; PI, plasma infusion; TR, treatment resistance.
